# *Pseudomonas aeruginosa* inhibits the growth of pathogenic fungi: *In vitro* and *in vivo* studies

**DOI:** 10.3892/etm.2014.1631

**Published:** 2014-03-21

**Authors:** LINGQING XU, FENG WANG, YIN SHEN, HONGYAN HOU, WEIYONG LIU, CAILIN LIU, CUI JIAN, YUE WANG, MINGYUE SUN, ZIYONG SUN

**Affiliations:** Department of Clinical Laboratory, Tongji Hospital, Tongji Medical College, Huazhong University of Science and Technology, Wuhan, Hubei 430030, P.R. China

**Keywords:** *Pseudomonas aeruginosa*, pathogenic fungi, inhibitory effect, cross-streaking method

## Abstract

The aim of the present study was to investigate the inhibitory effect of *Pseudomonas aeruginosa* (PA) on pathogenic fungi, including *Candida albicans* (CA), *Candida tropicalis* (CT), *Candida glabrata* (CG), *Candida parapsilosis* (CP) and *Candida krusei* (CK), *in vitro* and *in vivo*. In total, 24 PA strains were collected from clinical specimens and identified by Gram staining, oxidase production and the API 20NE system. Cross-streak, disk diffusion and co-culture methods were used to observe the inhibitory effect of PA. Sodium dodecyl sulfate-polyacrylamide gel electrophoresis was used to analyze differences in the bacterial proteins of PA. A blood infection model in mice was used to evaluate the effect of PA on fungi *in vivo*. The *in vitro* and *in vivo* results demonstrated that a number of PA isolates exhibited a marked inhibitory effect on pathogenic fungi, including CA, CT, CP, CG and CK, while other PA strains exhibited no effect. Therefore, PA exhibits an inhibitory effect on pathogenic fungi and this activity may be important in the treatment of patients. It was hypothesized that PA secretes various types of proteins to suppress the growth of fungal filaments, which subsequently inhibits pathogenic fungi.

## Introduction

Microbial natural products have been the source of the majority of antibiotics that are currently used for the treatment of various infectious diseases. Since penicillin was identified in 1928, studies on bacteria and fungi have revealed that microorganisms are a rich source of structurally unique bioactive substances (1). Following penicillin, numerous other drugs, including chlortetracycline, chloramphenicol, streptomycin, erythromycin, rifamycin, lincomycin, cephalosporin C, vancomycin, nalidixic acid, amphotericin B, nystatin and daunorubicin, the antitumor agent, were identified from microorganisms (2).

At present, a number of the pathogens involved in infectious disease are rapidly developing resistance to the available antibiotics (3), making treatment of these infections challenging (4). Therefore, research into more effective antibiotics is required.

Pseudomonads represent the major group of non-differentiating microorganisms that produce antibiotics. The antibiotic substances produced by this group of organisms are pyocyanin, pyrrolnitrin and pseudomonic acid (5,6). Previous studies have reported that *Pseudomonas aeruginosa* (PA) in clinical strains exhibit antifungal activity. In addition, in cystic fibrosis (CF) patients infected with PA, the occurrence of fungal infections is rare (7–9). These phenomena demonstrate that PA may exhibit antifungal activity. In the present study, the association between specific pathogenic fungi, including *Candida albicans* (CA), and PA was described, with the aim of investigating the mechanism behind the lethal and inhibitory effects that PA exhibits on fungi *in vitro* and *in vivo*.

## Materials and methods

### Strains

In total, 24 non-repetitive strains of PA (from various specimens: tracheal aspirate 20.8% (5/24); urine 16.6% (4/24); wound 25% (6/24); sputum 25 % (6/24); blood 12.5% (3/24); were obtained from various specimens at the in-patient department at Tongji Hospital (Wuhan, China) between May and September 2012. The strains were identified by Gram staining, the oxidase test and a Vitek-2 automated microbial identification system (bioMérieux, Inc., Craponne, France), or by the API 20NE system (bioMérieux, Inc.). All PA strains produce pyocyanin pigment. *Escherichia coli* [23922; American Type Culture Collection (ATCC), Manassas, VA, USA], *Klebsiella pneumoniae* (ATCC 700603) and PA (ATCC 25923) were maintained as quality control strains. The five strains of candida were collected from clinical specimens (*Candida albicans*, *Candida tropicalis*, *Candida glabrata* and *Candida krusei* were from sputum, *Candida parapsilosis* from urine) and were identified by CHROMagar Candida plates (CHROMagarCompany, Paris, France) and API-20C AUX yeast-like fungus identification strips (bioMérieux, Inc.). CA (ATCC 90028) was preserved as the control strain. The isolated PA strains were stored at 4°C on nutrient agar slopes, while the *Candida* isolates were stored on Sabouraud dextrose agar (SDA; Oxoid Ltd., Basingstoke, UK) plates until required for study.

### Animals

BALB/c mice were purchased and maintained separately at the Animal Facility of Tongji Medical College, Huazhong University of Science and Technology (Wuhan, China) under controlled conditions (specific pathogen free, 22°C, 55% humidity, and 12 h light/dark cycle). All experimental procedures on animals used in this study were performed under a protocol approved by the Institutional Animal Care and Use Committee at the Tongji Medical College. Thirty mice were purchased and were used in this experiment. Male mice (age, 8–10 weeks; weight, 25–30 g) were selected for the study. All experimental procedures on animals used in the study were performed under a protocol approved by the Institutional Animal Care and Use Committee of Tongji Medical College.

### Disk diffusion method

Fungi (400 μl; 1×10^8^ cells/ml) were spread on SDA plates (one plate was used for each fungi species) using a glass spreader and sterile filter paper disks were placed on the plates. Each PA strain was grown overnight at 37°C in Luria-Bertani (LB) media. Next, 3-μl specimens of the PA cultures (8×10^9^ CFU/ml) were spotted on the filter disks, which was followed by incubation at 30°C for up to 48 h. Cultures of same volume and concentration of *Escherichia coli* (ATCC 23922), *Klebsiella pneumoniae* (ATCC 700603), *Pseudomonas aeruginosa* (ATCC 25923) and sterile water were respectively spotted on the central filter disks as a control.

### Cross-streak method

Cross-streaking was performed according to the method described by Kerr (10). A fresh 24-h plate culture of each PA strain (1×10^8^ CFU/ml) was prepared as an inoculum in 0.9% NaCl to be tested for antifungal activity. The inoculum (30 μl) was streaked diametrically at a width of 1 cm across the SDA and blood agar (BA). SDA was used since it enhances fungal growth and all the tested strains of *Pseudomonas* were shown to grow well on the substance. The plates were incubated at 30°C for 24 h and the macroscopic growth was then removed from the plate using a sterile glass slide.

Sterile filter paper disks (diameter, 5 cm) were soaked in chloroform and laid on a metal tray in a safety cabinet. Each plate was then placed face down, without the lid, on top of a chloroform-containing filter paper disk; the plates were left for 30 min in order to kill the microscopic remnants of the culture. The plates were then removed from the cabinet and traces of chloroform were eliminated by exposure to air for a few minutes. A fresh 24-h plate culture of each fungal strain was used to prepare an inoculum of 1×10^6^ CFU/ml. This fungal suspension was streaked onto the chloroform-treated medium at right angles to the line of the original inoculum and the plates were incubated for 24 h at 30°C. Each of the 24 PA strains was tested against each of the five fungal strains and total inhibition of fungal growth was recorded as (+), partial inhibition of fungal growth was recorded as (±) and no inhibition of fungal growth was recorded as (−).

### Fungal strains co-cultured with PA

Sterile eppendorf tubes (EPs) were filled with 1 ml LB and each aforementioned PA and fungal suspension (50 μl) was added. Each fungal suspension (50 μl) was also added as a control. The EPs were agitated at 100 rpm at 30°C for 48 h.

### Sodium dodecyl sulfate-polyacrylamide gel electrophoresis (SDS-PAGE) analysis of the differences in PA bacterial protein

PA1206 and PA1215 strains, which exhibit a strong inhibitory effect on fungi, as well as the PA1201 and PA1222 strains, which present no inhibitory effect, were analyzed with SDS-PAGE. The four strains of PA were cultured in EPs containing 1 ml LB under conditions of 100 rpm at 30°C for 24 h (two replicates for each PA strain were prepared). The two replicates were boiled at 100°C for 10 min, then centrifuged at 14,500 × g for 1 min and the supernatant and sediment were extracted for SDS-PAGE. The steps of SDS-PAGE were performed according to Thermo Fisher Scientific Inc. (Waltham, MA, USA). The results were observed following bleaching.

### Blood infection in mice

A model of blood infection was applied to evaluate the anticandidal activity of PA in mice. PA and *Candida* species are often identified on skin and in the mucosa of healthy individuals. When the host defenses falter, PA and *Candida* initiate invasive growth that leads to severe diseases. BALB/c mice weighing 25–30 g were used in this study. All animals received humane care. The mice were randomly assigned to the following three groups. Group 1 was the total inhibition positive group (n=5+5), where five mice applied with PA1206 and five mice applied with PA1215 were tested against CA (ATCC 90028). Group two was the no inhibition group (n=5+5), where five mice applied with PA1201 and five mice applied with PA1222 were tested against CA (ATCC 90028). Finally, group three was the control group (n=10) and only CA (ATCC 90028) was applied. The bacterial and yeast suspensions (0.2 ml; 1×10^8^ CFU/ml) were injected into the caudal vein of the mice in groups one and two, while only the yeast suspension (0.2 ml; 1×10^8^ CFU/ml) was injected into the caudal vein of the mice in group three. At 24 h after the injections, blood samples were collected from the mice by the method of retro-orbital blood collection. The blood drops were incubated in BA and SDA at 30°C for 48 h, and the growth of the PA and CA strains was evaluated.

## Results

### Disk diffusion method

**Figure 1:**
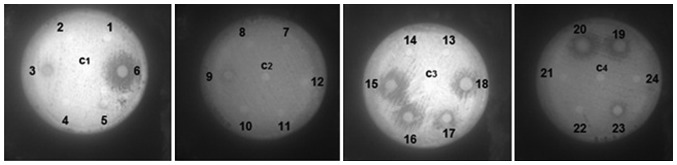
Disk diffusion tests. 1-24, PA1201-24; C1, *Escherichia coli* (ATCC 23922); C2, *Klebsiella pneumoniae* (ATCC 700603); C3, *Pseudomonas aeruginosa (*ATCC 25923); C4, sterile water. PA, *Pseudomonas aeruginosa*; ATCC, American Type Culture Collection.

PA strains 1206, -15, -16, -17, -18, -19 and -20 demonstrated clear zones of inhibition, while strains 1203, -09 and -23 presented partial zones of inhibition that were not very large. The remaining strains and the control strain exhibited no inhibition. Diameters of the zones of inhibition were measured; (−) no zone of inhibition; (±) zone of inhibition: 7–10 mm; (+) zone of inhibition: >10 mm (Fig. 1 and Table I).

### Cross-streak method results

**Figure 2:**
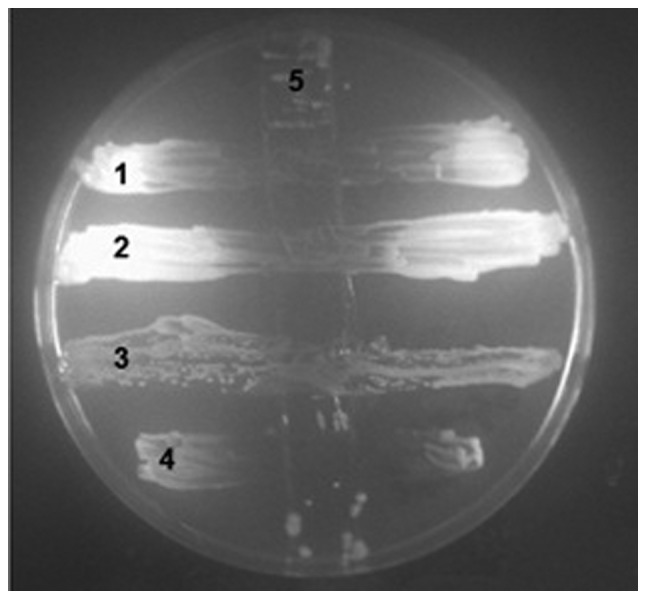
Cross-streak method was performed on 1, CA (ATCC 90028); 2, CT; 3, CG; 4, CP; 5, PA1206 following killing by chloroform. CA, *Candida albicans*; CT, *Candida tropicalis*; CG, *Candida glabrata*; CP, *Candida parapsilosis*; PA, *Pseudomonas aeruginosa*; ATCC, American Type Culture Collection.

The cross-streak method results were consistent with those of the disk diffusion method (Fig. 2).

### Fungi co-culture with PA

**Figure 3:**
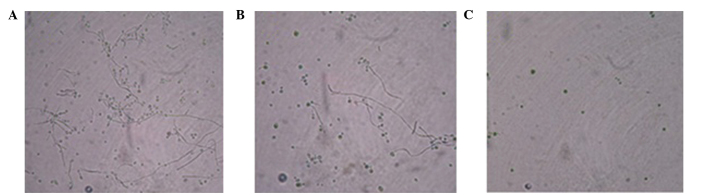
CA co-culture with PA. (A) CA was cultured alone in LB for 24 h and a huge number of fungal spores and hyphae growing are visible. (B) CA was co-cultured with PA1222 in LB for 24 h and numerous fungal spores and hyphae growing are visible. (C) CA was co-cultured with PA1206 and a few spores are visible, however, there are almost no hyphae growing. CA, *Candida albicans;* PA, *Pseudomonas aeruginosa*; LB, Luria-Bertani.

When the fungi were co-incubated with known inhibitory PA strains, a significant reduction in the production of fungal hyphae was observed. However, when the fungi were co-incubated with non-inhibitory PA strains, the production of fungal hyphae was similar to that of fungi that had been cultured alone in LB medium (Fig. 3).

### SDS-PAGE analysis of bacterial protein differences

**Figure 4:**
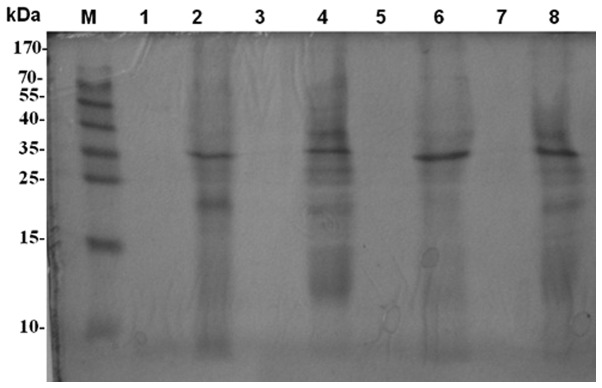
SDS-PAGE. M, protein marker; 1, PA1201 supernatant; 2, PA1201 sediment; 3, PA1206 supernatant; 4, PA1206 sediment; 5, PA1215 supernatant; 6, PA1215 sediment; 7, PA1222 supernatant; 8, PA1222 sediment; SDS-PAGE, sodium dodecyl sulfate-polyacrylamide gel electrophoresis; PA, *Pseudomonas aeruginosa*.

The two replicates of each PA strain produced the same pattern. The supernatants produced few bands, but the sediments presented the same bands and a number of different bands. Almost all the strains had one band in common at ~35 kDa. However, the inhibitory PA strains (1206 and 1215) produced distinct strips at 38, 35, 27 and 24 kDa, while the non-inhibitory PA strains (1201 and 1222) presented no bands at those points according to the marker. These observations indicate that the PA1206 and PA1215 strains secreted a greater variety of proteins compared with the PA1201 and PA1222 strains. An association between these bands and the growth inhibition effect on pathogenic fungi may exist, however, this requires further study (Fig. 4).

### Blood infection in mice

The mouse model of blood infection with CA and PA revealed that in group 1, no yeast was recovered from the infected mice, despite PA having 100% detection. In group 2, PA and yeast were recovered on the plate, while in group 3, 100% yeast was recovered.

## Discussion

Numerous antimicrobial compounds have been isolated from microorganisms. Microbial natural products are the source of the majority of antibiotics that are used currently for the treatment of various infectious diseases (2), including fungal infection. Techniques to identify novel products against pathogenic fungi have become increasingly important in the field of infection.

Interactions between prokaryotes and eukaryotes are ubiquitous. Despite the fact that the pathogenic and symbiotic associations that bacteria have with plants and animals have garnered the most attention, the prokaryote-eukaryote encounters that occur among microbes are likely to be far more common (11). Bacteria and unicellular eukaryotes, including yeasts and filamentous fungi, are found together in a myriad of environments and exhibit synergistic and antagonistic interactions

Previous studies have demonstrated that an interaction exists between PA and a number of other pathogenic fungi in the human body. Hughes and Kim (7) demonstrated that in CF patients infected with PA, only 10% of patients produced positive CA skin tests compared with 30% positivity in those free of PA, indicating that the antifungal substance produced by PA prevents *Candida* infections. There are also studies investigating the growth inhibition effect of PA in *Cryptococcus* species (12,13). However, to the best of our knowledge, there have been no studies regarding the isolation of *Cryptococcus* species from patients with CF. Considering that *Cryptococcus* and PA are common lung pathogens, the lack of co-colonization may result from the antifungal effect of PA on the growth of *Cryptococcus neoformans*.

Grillot *et al* (14) investigated the interactions between PA and yeast following incubation with a number of pure and mixed cultures. The authors demonstrated that the growth of all the isolates tested was inhibited by PA in blood culture medium and in bacterial culture filtrate.

Hogan and Kolter (11) described the pathogenic interaction between PA and CA. PA forms a dense biofilm on CA filaments and kills the fungus. Several PA virulence factors, including type IV pili, phospholipase C and phenazines, that are important in disease are also involved in the killing of CA filaments. Pyocyanin and *Pseudomonas* quinolone signal accumulate intensively in the lung mucus of patients with CF (8,9); these antifungal molecules may be important in the prevention of pulmonary cryptococcosis in patients with CF.

Fungal-bacterial interactions also occur in skin and nail infections. Foster *et al* (15) observed that dermatophyte and non-dermatophyte fungi grew poorly in the presence of PA, and demonstrated that large PA populations in infected nails resulted in a lower fungal population.

In the present study, the disk diffusion and cross-streak methods produced similar results demonstrating the lethal and inhibitory effects of PA on *Candida* species. The results revealed that certain PA strains exhibit a strong antifungal ability, while others exhibit partial or no ability. In the co-culture of PA with pathogenic fungi in LB medium, the *Candida* species produced a markedly large number of filaments when cultured alone or with non-inhibitory PA strains. However, when the fungi were cultured with inhibitory PA, almost no filaments were produced.

SDS-PAGE revealed that PA1206 and PA1215 secreted different proteins from those of strains 1201 and 1222), yet PA1206 and 1215 exhibited similar secreted protein patterns. PA1201 and 1222 also showed a similar secretion pattern. All the strains had one band in common at ~35 kDa. However, the inhibitory PA strains (1206 and 1215) produced distinct strips at 38, 27 and 24 kDa, while the non-inhibitory PA strains (1201 and 1222 ) presented no bands at those points. PA1206 and 1215 exhibit strong inhibitory effects on pathogenic fungi, while PA1201 and 1222 show no effects. Therefore, an association may exist between the difference of secreted proteins in the two types of strain and the inhibitory effect on pathogenic fungi; the proteins may be associated with the inhibitory effect on the growth of fungal filaments. However, the mechanism requires further study.

In the animal model of bacteremia, a suspension of PA and CA was injected into the caudal vein of the mice. After 24 h, blood drops were obtained using the method of orbital blood collection and they were incubated on BA and SDA. In group 1, the PA strains had been recovered, but no strains of CA were isolated. In group 2, PA and CA were observed. However, in the control group 3, 100% CA was recovered from the blood. These results are in accordance with a previous study of a rabbit model of concomitant fungemia with CA and bacteremia with PA, in which no yeast was recovered from the blood cultures despite 100% detection of PA (16). Therefore, the present study investigated the *in vivo* and *in vitro* antifungal activity of PA strains against *Candida* species. The results demonstrated that the inhibitory effect of PA exists *in vivo* and *in vitro*. To the best of our knowledge, the present study is the first study to use a mouse model of co-infection with PA and *Candida*. The present study investigated the *in vivo* anticandidal activity of PA in a mouse model of blood infection. The results demonstrated that PA strains inhibited the growth of *Candida* strains by 100%, which is in accordance with previous studies (17,18).

In conclusion, PA strains exhibit potent antifungal activity *in vitro* and *in vivo*. The underlying mechanism may involve pyocyanin, biofilms and the production of a variety of factors, including various types of proteins, a quorum sensing system, redox phenazin and phospholipase C, which inhibit fungal filaments and thereby inhibit fungal growth. Further study on the separation and purification of these substances is required to improve the treatment and prevention of fungal infections.

## Figures and Tables

**Table I:** Antifungal activity of PA on pathogenic fungi.

	PA strains																								Control strains		
		
Fungi	1	2	3	4	5	6	7	8	9	10	11	12	13	14	15	16	17	18	19	20	21	22	23	24	EC	KP	PA
CA	−	−	±	−	−	+	−	−	±	−	−	−	−	−	+	+	+	+	+	+	−	−	±	−	−	−	−
CT	−	−	±	−	−	+	−	−	±	−	−	−	−	−	+	+	+	+	+	+	−	−	±	−	−	−	−
CG	−	−	±	−	−	+	−	−	±	−	−	−	−	−	+	+	+	+	+	+	−	−	±	−	−	−	−
CP	−	−	±	−	−	+	−	−	±	−	−	−	−	−	+	+	+	+	+	+	−	−	±	−	−	−	−
CK	−	−	±	−	−	+	−	−	±	−	−	−	−	−	+	+	+	+	+	+	−	−	±	−	−	−	−

−, no inhibition; ±, partial inhibition; +, total inhibition of fungal growth. PA, *Pseudomonas aeruginosa* (ATCC 25923); EC, *Escherichia coli* (ATCC 23922); KP, *Klebsiella pneumoniae* (ATCC 700603); CA, *Candida albicans*; CT, *Candida tropicalis*; CG, *Candida glabrata*; CP, *Candida parapsilosis*; CK, *Candida krusei*; ATCC, American Type Culture Collection.
